# Complicated sternal dehiscence treated with the strasbourg thoracic osteosyntheses system (STRATOS) and the transposition of greater omentum: a case report

**DOI:** 10.1186/1749-8090-5-53

**Published:** 2010-06-28

**Authors:** Fabrizio Ceresa, Giuseppe Casablanca, Francesco Patanè

**Affiliations:** 1 Cardiac Surgery Division, Papardo Hospital, Messina, Italy; 2 Thoracic Surgery Division., Papardo Hospital, Messina, Italy

## Abstract

Sternal dehiscence is a serious complication after cardiac surgery. Sternal refixation, performed by simple rewiring or techniqual modification of rewiring as described by Robicsek, can fail, overall when the bone quality is poor or the sternum is completely destroyed. The sternal closure systems, consisting of plates, screws or rib clips and titanium bars, have been recently introduced to treat the complicated sternal dehiscence. We describe for the first time the use of the Strasbourg Thoracic Osteosyntheses System (STRATOS) and the greater omentum, to treat a complicated sternal dehiscence, causing chest pain and respiratory failure.

## Background

Middle sternotomy is the most common access to the heart and mediastinum used in the cardiac surgery. The incidence of sternal dehiscence with or without infection range from 0,5 to 5,0% and it is a serious complication after cardiac surgery. Obesity, osteoporosis, chronic obstructive pulmonary disease (COPD), diabetes, intake of corticosteroids and off-midline sternotomy are the main risk factors [1]. Sternal refixation can be performed by simple rewiring or techniqual modification of rewiring as described by Robicsek and colleagues [1]. When the bone quality is poor or there are multiple fractures of the sternum, these classical approaches of rewiring can fail and the rigid fixation systems, recently introduced, can be used for the sternal reconstruction [1,2]. We describe the case of a complicated sternal dehiscence, treated with the implant of the Strasbourg Thoracic Osteosyntheses System (STRATOS) and the transposition of the greater omentum.

## Case presentation

A 75 years-old man with COPD, diabetes, osteoporosis and recent onset of atrial fibrillation underwent aortic valve replacement for severe aortic valve stenosis, complicated by sternal dehiscence, treated by modified rewiring according to Robicsek's technique. The sternal dehiscence, depending on the fractures of some right ribs, occurred again after a week.

**Figure 1:**
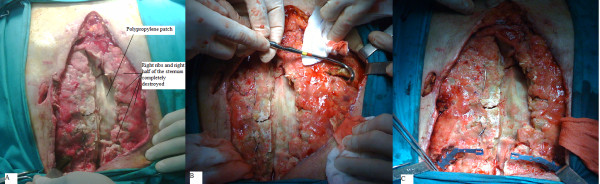
**The sternal wound is opened and the right half of the sternum is completely destroyed (A)**. The intercostal muscles and the bundle is removed (B). The rib clips are clung to the rib through "small handles" that are crimped with the rib clip fixation pliers (C).

After having implanted a polypropylene patch to avoid injuries to the underlying heart, we have initially used the vacuum assisted closure (VAC) device to stabilize the chest wall with the negative pressure produced and to sterilize the wound (Figure 1A).

When the wound was germe-free, we performed the sternal reconstruction, utilizing the STATOS.

The rib clips were crimped with the rib clip fixation pliers on the second, fourth and sixth ribs of the both sides, after having removed the intercostal muscles and the bundle. (Figure 1B and 1C).

**Figure 2:**
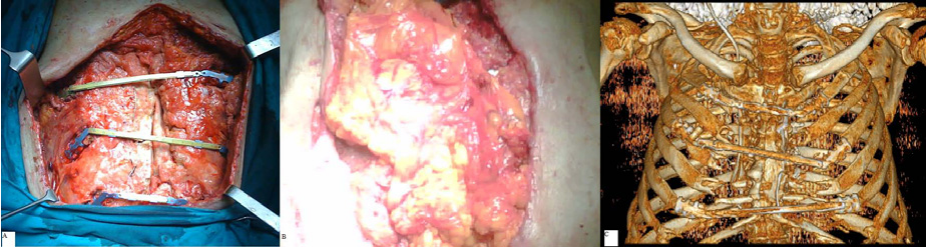
**The connecting bars are secured to the rib clips, positioned on the second, fourth and sixth ribs (A)**. The greater omentum has been used to cover the STRATOS fixation system (B). Three-dimensional computed tomography reconstruction showing the titanium rib bridges maintaining the chest stability (C).

The connecting bar was cut to the correct length and secured to the clip by crimping (Figure 2A).

The remaining dead space was filled with the greater omentum, transposed through the diaphragm and used to cover the titanium sternal closure system, facilitating the healing of the wound (Figure 2B)

The CT scan afterwards showed the correct position of the titanium rib bridges (Figure 2C). The patient was in hospital discharged after one month.

## Discussion

Sternal dehiscence with or without infection is a serious complication after sternotomy and it increases the mortality and morbidity rates [1].

When the bone is very osteopenic, the classical techniques of rewiring are associated with a high rate of recurrence dehiscence. New approaches of sternal reconstruction, based on the use of orthopaedic, plastic and maxillofacial fixation systems, have been recently introduced. The sternal closure systems, consisting of titanium reconstruction plates, cables and screws, perform a transverse rib-to-rib stabilization without the adhesiolysis of the substernal area, extending the zone of fixation beyond the fractured sternum to the ribs laterally, where the bone quality should be better [1,2].

We have carefully assessed what was the better sternal refixation system to use in our case.

The STATOS has been recently utilized to treat surgically the chest wall deformities as Pectum excavatum, to stabilize the fractures after severe thoracic trauma or to reconstruct the chest wall after removal of a tumoral mass [3].

Each implant consists of two rib clips straight united by a connecting bar. The three different angulations of the rib clips and the adjustable length of connecting bar allow to use this system for any anatomical situations.

The rib clip is clung to the rib through "small handles", that surround it without compressing the vasculo-nervous bundle. In this way the rib should be weakened less than by the bi-cortical insertion of the screws.

The remaining dead space after sternal reconstruction has been filled with the greater omentum. The use of the greater omentum in the treatment of the mediastinitis and deep sternal wound infection has been described in the literature and its immunological and angiogenetic properties can prevent the further extension of local infection and facilitate the primary closure of the superficial tissues, leading to the healing of the sternal wound [4].

In conclusion, we purpose the use of STRATOS, covered by the transposition of the greater omentum, to treat the complicated sternal dehiscence after sternotomy, particularly in those cases in which the bone quality is poor and the sternum is completely destroyed. This system is effectiveness in the stabilization of the chest wall, reducing the pain, improving the ventilation and facilitating the quick healing of the wound.

## Authors Contributions

Ceresa Fabrizio and Patane Francesco had written the case report. Casablanca Giuseppe had dealt the images. All authors read and approved the final manuscript.

## Competing interests

The authors warrant that no ethical problem or conflicts of interest regarding this paper exist.

## Consent

Written informed consent was obtained from the patient of this case report and accompanying the images. A copy of written consent is available for the review by the Editor in Chief of this journal

## References

[B1] Voss B Bauernschmitt R Albrecht W Krane M Kröss R Brockmann G Libera P Lange R Sternal reconstruction with titanium plates in complicated sternal dehiscence Eur J Cardiothorac Surg 2008 34 139 145 10.1016/j.ejcts.2008.03.030 18455410

[B2] Huh J Bakaeen F Chu D and Wall MJ Transverse sternal plating in secondary sternal reconstruction J Thorac Cardiovasc Surg 2008 136 1476 1480 10.1016/j.jtcvs.2008.03.051 19114193

[B3] Coonar AS Qureshi N Smith I Wells FC Reisberg E Wihlm JM A novel titanium rib bridge system for chest wall reconstruction Ann Thorac Surg 2009 87 e46 e48 10.1016/j.athoracsur.2009.01.069 19379855

[B4] Moreschi AH Vieria de Maredo Neto A Barbosa GV Saueressig MG Aggressive treatment using muscle flap or omentopexy in infections of the sternum and the anterior mediastinum following sternotomy J Bras Pneumol 2008 34 9 654 660 10.1590/S1806-37132008000900004 18982201

